# Implant removal using thermal necrosis—an in vitro pilot study

**DOI:** 10.1007/s00784-020-03361-x

**Published:** 2020-06-04

**Authors:** Kristian Kniha, Eva Miriam Buhl, Benita Hermanns-Sachweh, Faruk Al-Sibai, Anna Bock, Florian Peters, Frank Hölzle, Ali Modabber

**Affiliations:** 1 Department of Oral and Cranio-Maxillofacial Surgery, RWTH Aachen University, Pauwelstraße 30, 52074 Aachen, Germany; 2 Institute of Pathology, Electron Microscopy Facility, RWTH Aachen University, Aachen, Germany; 3 Private Institute for Implant Pathology, ZBMT, Campus Melaten, Pauwelsstaße 17, 52074 Aachen, Germany; 4 Institute of Heat and Mass Transfer, RWTH Aachen University, Augustinerbach 6, 52056 Aachen, Germany

**Keywords:** Matrix degeneration, Osteocyte, Temperature, Histopathology, SEM

## Abstract

**Objectives:**

The purpose of this pilot porcine cadaver study was to evaluate the feasible temperature thresholds, which affect osteocyte viability and bone matrix in a preclinical setup, assessing the potential of thermal necrosis for implant removal for further in vivo investigations.

**Materials and methods:**

After implant bed preparation in the upper and lower jaw, temperature effects on the bone were determined, using two tempering pistons with integrated thermocouples. To evaluate threshold temperature and time intervals leading to bone necrosis, one piston generated warm temperatures at 49 to 56 °C for 10 s and the other generated cold temperatures at 5 to 1 °C for 30 s. Effects were assessed by a semi-quantitative, histomorphometrical scoring system, scanning electron microscopy (SEM), energy-dispersive X-ray spectroscopy (EDX), and transmission electron microscopy (TEM).

**Results:**

The bone matrix was significantly degenerated starting at 51 °C for 10 s and 5 °C for 30 s. The osteocyte condition indicated significant bone damage beginning at cold temperatures of 2 °C. Temperature inputs starting at 53 °C led to decalcification and swollen mitochondria, which lost the structure of their inner cristae.

**Conclusions:**

This study identified temperatures and durations, in both heat and cold, so that the number of samples may be kept low in further studies regarding temperature-induced bone necrosis. Levels of 51 °C for 10 s and 5 °C for 30 s have presented significant matrix degeneration.

**Clinical relevance:**

Temperature thresholds, potentially leading to thermo-explantation of dental implants and other osseointegrated devices, were identified.

## Introduction

Dental implants, replacing missing teeth, offer a therapeutic option, which has become indispensable in modern dentistry.^1^ As with any therapy, however, complications can occur. The worst-case scenario occurs when it becomes necessary to remove osseointegrated implants.^2^ Reasons for this extreme measure can include implant fractures, wear and tear resulting in fitting inaccuracies, inflammation (peri-implantitis), and esthetic problems.^3^ Currently, in the absence of these complications, osseointegration is considered an irreversible process, as no technically predictable way exists to reverse the growth of bone cells on the implant surface. In most cases, implant removal is simple, and only a small percentage of implants cannot be easily extracted.^4^ Implants with severe bone resorption are usually much simpler to remove than implants that are completely integrated into the bone, such as implants with incorrect positioning.^5^ Traumatic implant removal further complicates new implant rehabilitation.^5^ The counter torque ratchet technique is one of the most atraumatic ways of explanting—preserving valuable surrounding bone.^6^ This study, however, has specifically focused on an explantation method in which thermal bone necrosis is induced. This is an interesting subject because a relatively small percentage of implants cannot be easily extracted by a high torque wrench.^5^ Several case reports have shown that implants can be easily removed after an initial, uncontrolled random thermal treatment, which results in osteonecrosis of the jaw.^7–9^ The authors’ aim, therefore, was to heat the implant in such a way that peri-implant bone necrosis was induced, with subsequent implant loosening. Data are scarce in the current literature supporting using a controlled thermo-explantation procedure or indicating the precise temperature and time interval that would produce sufficient, but minimal, osteonecrosis around the implants to remove them without incurring trauma. High and uncontrolled temperatures could easily lead to extensive jaw necrosis and, instead of an atraumatic explantation, would, rather, result in severe inflammation. Consequently, for successful thermo-explantation, a precise temperature must be selected within the initial developmental range of bone necrosis.

Usually, thermal bone damage during surgical interventions is an undesirable side effect, which may lead to bone damage. Several studies have presented threshold values of warm temperatures that will induce bone necrosis. For example, Rouiller (1953) published a threshold value of 55 °C and a duration of 1 min^10^; Lundskog (1972) showed lower values of 50 °C and 30 s,^11^ while Erikssen and Albrektsson (1983) used 47 °C and 1 min^12^. By contrast, Goetz et al. examined cryoinsult and found that cold temperatures could also cause osteonecrotic lesions in bone tissues.^13, 14^ They concluded that temperatures below 1–3.5 °C produced histologically confirmed bone necrosis. These previous studies indicate that the temperature thresholds for osteonecrosis in the current literature show major deviations.

Next to the unclear threshold levels that lead to jaw necrosis, osteonecrosis itself is not a specific disease entity but is, instead, the final common pathway of several conditions that lead to bone death.^15^ Depending on the temperature, the bone–implant interface may show hyperemia, necrosis, fibrosis, osteocyte degeneration, or bone resorption. In addition, biopsy material from affected areas shows partially or completely necrotic bone, with empty osteocytic lacunae and heavily eroded surfaces.^15^

The primary aim of this cadaveric pilot study was to evaluate a feasible temperature threshold that affects the osteocyte cells and the jawbone matrix in the upper and lower jaw using both warm and cold temperatures. The authors hypothesized that an appropriate temperature/time interval for warm and cold temperatures leads to a minimal appearance of dead osteocytes and matrix degeneration.

## Material and methods

**Fig. 1:**
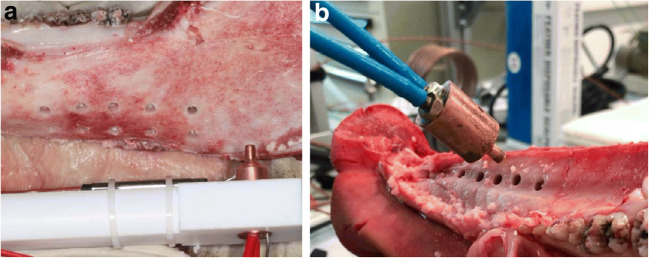
**a** Example of the electric tempering piston generating warm temperatures in the drilled holes of the porcine mandible. **b** Example of the water-cooled tempering piston for the drilled holes of the porcine maxilla

In this pilot study, two tempering pistons, capable of generating warm or cold temperatures with integrated thermocouples, were developed at the Institute of Heat and Mass Transfer of the University. Warm temperatures were generated by an electric wire device, and cold temperatures were generated with a device incorporating a cooled water circuit (Fig. 1).

At first, based on the current literature, to limit the possible temperatures and intervals leading to bone necrosis and to check the workflow, an initial test series was carried out at temperatures of 1 to 7 °C for 30 s and of 38 to 58 °C for 10 s^10,11,13,14^ (*n* = 1 for each temperature). This test sample was histopathologically evaluated to define the extent of the temperature/time interval. The sample showed detectable dead osteocyte lacunae at 52 °C and 10 s and at 3 °C and 30 s. Based on this information, the temperature range for the assessment was set at 49 to 56 °C for 10 s and 5 to 1 °C for 30 s.

Subsequently, the main investigation was conducted. Therefore, a total of 14 pig heads and 140 drill samples (group size of ten samples) were analyzed for thermal bone damage in this histomorphometric analysis. For each temperature group, ten samples were evaluated—five samples from the upper jaw and five samples from the lower jaw.

Scanning electron microscopy (SEM), energy-dispersive X-ray spectroscopy (EDX), and transmission electron microscopy (TEM) analysis included a total of 18 drill samples. For each temperature, six samples were evaluated—three from the upper jaw and three from the lower.

The pork jaws were collected during the local slaughterhouse process and were integrated into the experiment 20 min later. The samples were collected in the morning; the assessment was carried out at the university clinic; and, afterwards, the samples were immediately stored in prepared tubes, with a maximum time between slaughter and storage of 6 h. The included animals had a mean age of 7 months.

The laboratory temperature was maintained at 20 °C using thermostatic temperature control. Thermal data from the devices were simultaneously transferred and controlled by a computer. One surgeon carried out the drilling under strict cooling conditions, beginning with a pilot drill (diameter 2 mm, depth 8 mm), followed by drills with diameters of 3.3 mm and 3.8 mm (Camlog GmbH, Screw-Line, Wimsheim, Germany). Temperature damage during drilling and processing was ruled out by conducting control drillings without temperature treatment.

The pistons were fabricated with a 3.8 mm diameter and individual profile to fit in the drilled holes without pressure and with a congruent contact area to the wall (Fig. 1). The drilled holes had a sufficient diameter to allow the temperature device to show a clamping fit inside. Block samples of every drill were taken (Cut grinder, Exact GmbH, Norderstedt, Germany) to evaluate the walls of the drill holes. The sample assessment aimed to assess the depth of the drilled holes as close as possible to the center of 4 mm depth.

### Histomorphometric analysis

The samples were stored in 4% formalin (neutrally buffered with methanol) for 48 h (Otto Fischar GmbH & Co. KG, Saarbrücken, Germany). Decalcification was conducted for approximately 4 weeks at 37 °C by storing the samples in a 20-fold volume of ethylenediaminetetraacetic (EDTA, MolDecalcifer, Menarini, Florence, Italy). The EDTA solution was changed every 2 days. After a rinse with tap water, the samples were stored for 24 h in 5% sucrose with phosphate-buffered saline PBS (100 ml PBS, 5 g sucrose). The samples were then shock frozen in liquid nitrogen and embedded (TissueTek, Sakura, Alphen, Netherlands). Subsequently, cryostat sections of 5–7 μm thickness were cut, mounted on super frost slides, and dried. The samples were fixed in acetone for 10 min and then stained with Hematoxylin-Eosin (HE) and Ladewig stains, according to routine protocols. One specialized pathologist analyzed the tissue structures by light microscopy. The set of parameters considered in the histomorphometric analysis included a semiquantitative evaluation^16^ of the osteocyte condition (0 = normal osteocyte lacunae; 1 = minimal count of dead osteocytes; 2 = progressing count of dead osteocytes; 3 = high number of dead osteocytes) and tissue matrix degeneration (0 = normal bone tissue; 1 = minimal matrix degeneration; 2 = progressing matrix degeneration; 3 = severe matrix degeneration). In addition, the tissue fraying around the drill holes was measured quantitatively in micrometer, following representative tissue probes at each temperature. All parameters were examined under × 40 to × 200 magnification with a BX43 microscope equipped with a SC180 camera and cellSens Standard Version 1 software (Olympus Hamburg, Germany). For conducing measurements, only correctly calibrated images were used. (Images acquired with this software are automatically calibrated when the used objective is specified.) The outcome assessment was blinded.

### SEM, EDX, and TEM analyses

The SEM, ECX, and TEM analyses were conducted, in three iterations per analysis, at 50 °C, 53 °C, and 56 °C. The samples were fixed in 3% glutaraldehyde with 0.1 M Sorensen’s phosphate buffer, dehydrated in an ascending ethanol series (30–100%), and dried at 37 °C (SEM and ECX). The samples were then analyzed using an environmental scanning electron microscope (ESEM XL 30 FEG, FEI, Eindhoven, Netherlands) in a backscatter mode with an acceleration voltage of 15 kV. EDX analysis was performed with an EDAX Genesis System (EDAX, Mahwah, NJ, United States). SEM images in backscatter mode and EDX analyses were performed at five measurement points. SEM and EDX analyses were used to measure the element weights of carbon, oxygen, sodium, phosphate, and calcium.

For TEM, the samples were fixed in 3% glutaraldehyde with 0.1 M Sorensen’s phosphate buffer and decalcified in EDTA, as previously described. After post-fixation in 1% OsO4 (Roth, Karlsruhe, Germany) with 17% sucrose buffer, the samples were dehydrated in an ascending ethanol series, incubated in propylene oxide (Serva, Heidelberg, Germany), and embedded in Epon resin (Serva, Heidelberg, Germany). Ultrathin sections (70–100 nm) were cut and stained with 0.5% uranyl acetate and 1% lead citrate (both EMS, Munich, Germany) to enhance contrast. Samples were viewed at an acceleration voltage of 60 kV using a Zeiss Leo 906 (Carl Zeiss, Oberkochen, Germany) transmission electron microscope. TEM decriptivly evaluated the cellular structure, including cell organelles.

### Statistical analysis

The sample size was calculated using G*Power software (G*Power, Version 3.1.9.2, Düsseldorf, Germany).^17^ One-way ANOVA was used as an indication. The authors hypothesized that an individual temperature/time interval for warm and cold temperatures would lead to a minimal appearance of dead osteocytes and matrix degeneration. Using a 0.05 significance level, 14 groups, an effect size of 0.4 (based on the first test), a power of 80%,^18^ and a total of 126 samples were needed to verify the hypothesis.

Analyses were performed using the Prism 8 software for Mac OS X (GraphPad, La Jolla, CA, USA) running on Apple OS X. Variables were analyzed using the D’Agostino–Pearson normality test. The Kruskal–Wallis and Dunn’s multiple comparison tests (one-way ANOVA) were also used to identify the difference between the means of the subgroups. EDX analyses were compared using an unpaired *t* test. A *p* value of <0.05 was considered statistically significant. Spearman’s rho test was applied to evaluate the correlations between parameters. Values were considered to be “very weak” (0.00–0.19), “weak” (0.20–0.39), “moderate” (0.40–0.59), “strong” (0.60–0.79), or “very strong” (0.80–1.0).^19^

## Results

### Histomorphometric analysis

**Fig. 2:**
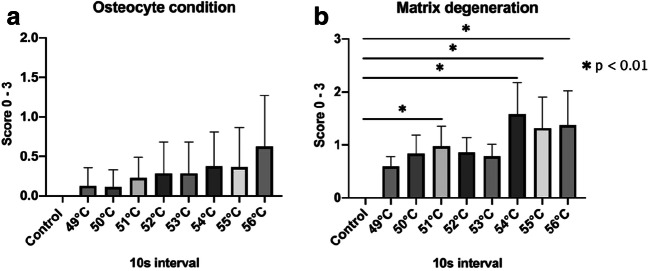
**a** Data evaluation of the osteocyte condition of the tissue in warm temperatures for 10 s intervals. **b** Data evaluation of the matrix degeneration in warm temperatures for 10 s intervals

The osteocyte condition indicated increasing bone damage starting at warm temperatures of 49 °C and rising to 56 °C; however, no significant differences were noted when compared with the control tissues (Fig. 2a). Even at 56 °C and 10 s, the number of dead osteocytes was minimal. By contrast, cold temperatures showed a significant difference in dead osteocyte counts between tissues treated at 1 °C and the control tissues, as well as between tissues treated at 1 °C and 2 °C for 30 s (Fig. 2b, *p* < 0.01). Cold temperatures, therefore, increased the count of dead osteocytes.

**Fig. 3:**
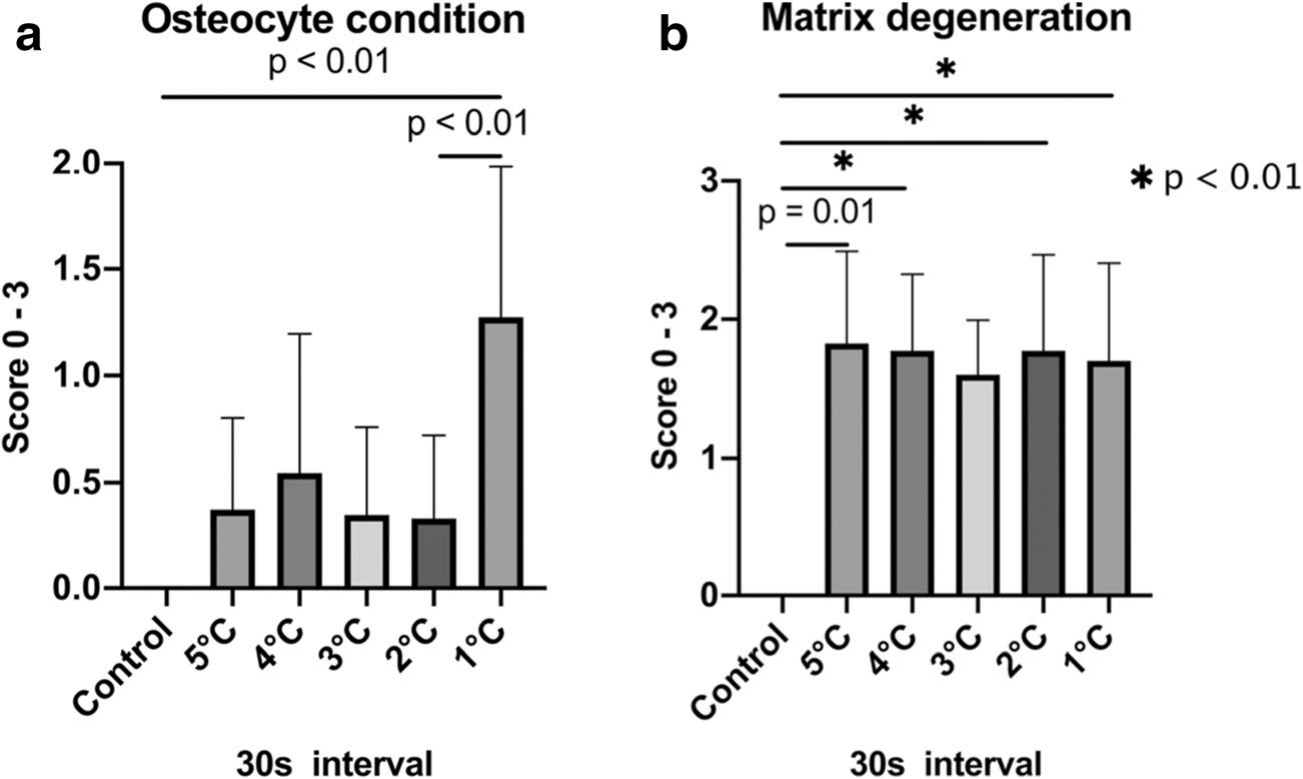
**a**, **b** Data evaluation of the osteocyte condition and matrix degeneration of the tissue in cold temperatures for 30 s intervals

**Fig. 4:**
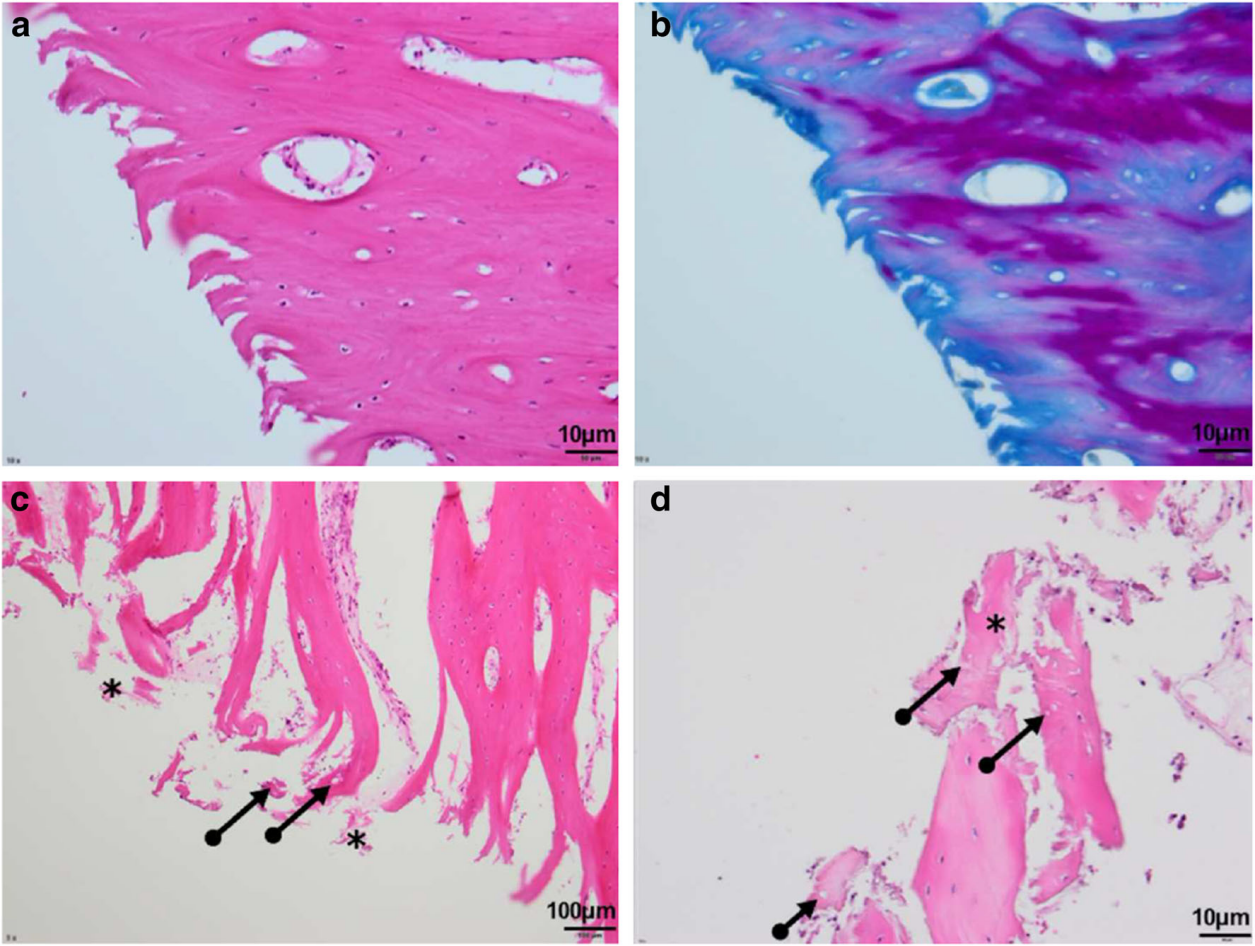
Temperature-dependent matrix degeneration with splicing of the bone matrix (**a** hematoxylin/eosin [HE] staining, × 200; **b** Ladewig staining, × 200). Necrotic cell detritus (asterisk) and empty osteocyte lacunae (arrow) (**c** HE staining, × 100; **d** HE staining, × 200)

Significant differences were observed for matrix degeneration of the hard tissue between the control tissues and those treated at 51 °C, 54 °C, 55 °C, and 56 °C for 10 s (Fig. 3a, *p* < 0.01). Similarly, all cold temperature treatments (except 3 °C) significantly increased the matrix degeneration when compared to the control group (Fig. 3b, *p* = 0.01 and < 0.01). The matrix degeneration was evaluated as more severe at the same temperature and time intervals as the osteocyte condition (Fig. 4).

The bone quality of the upper and lower jaw showed an insignificant, weak correlation between the osteocyte condition and warm temperatures (Spearman’s rho 0.31); it also showed an insignificant, moderate correlation for cold temperatures (Spearman’s rho 0.56, Table 1). The bone quality of either the upper or lower jaw showed an insignificant, weak correlation between the matrix degeneration and either cold or warm temperatures (Spearman’s rho 0.35 and 0.33, respectively; Table 1). The noticeable tissue fraying could not be correlated with temperature damage.

**Table 1:** Correlation values between parameters

Correlation	Spearman rho	*p* value
Between osteocyte condition at 49 to 56 °C and the upper or lower jaw	0.31	0.32
Between matrix degeneration at 49 to 56 °C and the upper or lower jaw	0.33	0.33
Between osteocyte condition at 5 to 1 °C and the upper or lower jaw	0.56	0.07
Between matrix degeneration at 5 to 1 °C and the upper or lower jaw	0.35	0.27
Between tissue fraying and the temperatures of 49 to 56 °C	0.24	0.69
Between tissue fraying and the temperatures of 5 to 1 °C	0.14	0.80

### SEM, EDX, and TEM analyses

**Fig. 5:**
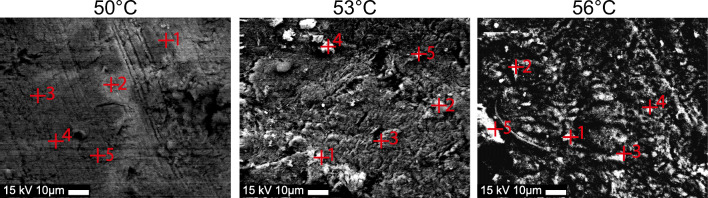
SEM images in backscatter mode and EDX analyses at 5 measurement points. At 50 °C, no bone debris development was evaluated. At 53 °C and 56 °C, debridement was analyzed and compared with the background

Eighteen total samples were included. The SEM images in backscatter mode and the EDX analyses revealed an irregular shape of the bone surface, with bone debris and signs of thermal damage. At 50 °C, no bone debris development was detected (Fig. 5).

At 53 °C and 56 °C, the extent of debridement was analyzed and compared to the background level. The ratio of calcium to phosphate (Ca/P) increased from 2.29 SD 0.27 (50 °C) to 3.86 SD 2.47 (56 °C), and the ratio of calcium to carbon (Ca/C) increased from 1.81 SD 0.04 (50 °C) to 2.13 SD 0.39 (56 °C). By contrast, the background showed decreasing values for the Ca/P and Ca/C ratios (Table 2).

**Table 2:** Descriptive and statistical values for energy-dispersive X-ray spectroscopy (EDX) analysis of thermal debris development. Debridement was assessed at 53 °C and 56 °C and compared with the background

		50 °C		53 °C			56 °C			*p* value
	Element	Mean	SD	Mean	SD	Background	Mean	SD	Background	
Weight %	Carbon	11.86	1.00	11.00	3.36	10.14	10.54	4.38	12.58	
	Oxygen	44.10	3.23	32.84	4.41	34.87	39.61	8.54	45.00	
	Sodium	2.04	0.11	1.08	0.15	1.58	2.03	0.45	2.61	
	Phosphate	14.98	1.42	17.57	1.49	17.51	15.07	2.57	14.01	
	Calcium	27.02	2.02	37.51	5.72	35.90	32.75	10.55	25.80	
Ratios	Ca/C	2.29	0.27	3.87	1.94	3.55	3.86	2.47	2.05	n.s.
	Ca/P	1.81	0.04	2.13	0.16	2.05	2.13	0.39	1,84	50–53 *p* < 0.01

**Fig. 6:**
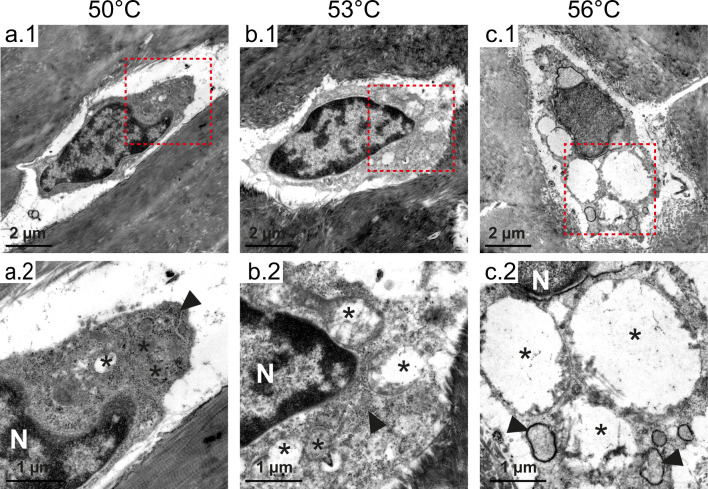
Transmission electron microscopy images of osteocytes (rectangle in **a**–**c**; 1, indicates the respective magnified area shown **a**–**c**; 2. N, nucleus; asterisks, mitochondria; arrowhead, endoplasmic reticulum). With increasing temperature, the osteocytes show typical signs of cellular stress. Beginning at 53 °C, the mitochondria are swollen and lose the structure of their inner cristae. At 56 °C, the lumina of the endoplasmic reticulum are pronouncedly dilated

Analysis of the cellular stress levels showed differences between the 50 and 53 °C treatments and between the 53 and 56 °C treatments (Fig. 6). After the 53 °C treatment, the osteocytes showed signs of cellular stress, such as swollen mitochondria and structure loss of the inner mitochondrial cristae. These mitochondrial changes were also observed with the 56 °C treatment, but the lumina of the endoplasmic reticulum were also pronouncedly dilated.

## Discussion

It has been reported that high-frequency surgical devices and dental lasers lead to unwanted thermal damage of peri-implant bone and may result in implant loss.^20, 21^ Such uncontrolled heating may also cause severe inflammation and jaw necrosis.^21^ However, several publications have described the successful loosening of osseointegrated implants using high temperatures with, for example, ultra-high frequency surgical devices.^8, 9^ In these studies, the heat input was intentional but was still uncontrolled and uneven. Nevertheless, this approach, using minimal thermal necrosis for implant removal, may preserve valuable bone tissue in difficult explantations.

The aim of this pilot study was, therefore, to evaluate the threshold temperature/time levels that would lead to minimum damage of osteocytes and bony tissue, as the eventual in vivo application of the protocol would require that peri-implant bone necrosis be kept at a minimum. In this cadaveric study, warm and cold temperature thresholds, which affect the viability of osteocyte cells and the structure of the jawbone matrix, were assessed. The available medical literature confirms that bone cells can be damaged by temperatures above 42 °C.^22^ For example, Eriksson and Albrektsson investigated the effects of heat on bone metabolism.^12^ Using animal experiments, they established a model in which a fixed heat chamber was used to record bone metabolism at different temperatures. This model allowed them to make statements about high/short versus low/long temperature treatments on bone cell metabolism. They found that the degree of damage depended on both the temperature and the exposure time. The critical value for irreversible bone damage was defined as 47 °C with an exposure time of 1 min.^12^ In general, short duration/high temperature applications (e.g., 50 °C for 1 min) had similar effects, on hard and soft tissue, to those effects observed with longer duration/lower temperature treatments (e.g., 47 °C for 5 min).^22^

A similar effect to that of heating can be achieved with targeted cooling. A single, cold stimulus between – 10 and – 20 °C inevitably leads to surrounding skin and bone necrosis. Even temperatures around 1 °C produce a histologically proven bone change in the surrounding tissue (max. 0.7 mm).^13, 14^

The present authors’ results indicated that 51 °C for 10 s and 5 °C for 30 s caused irritation that led to significant matrix degeneration; thus, these temperatures/durations may be the threshold levels for in vivo bone necrosis. Bone fraying does not appear to be caused by temperature treatment. Further research, however, should concentrate on in vivo processes, such as blood flow and bone remodeling, which may affect the threshold values of osteonecrosis.

Bone tissue is a heterogeneous structure with cancellous and cortical areas, which affect the individual temperature threshold. According to Trisi et al., during a 2-month follow-up period, the cancellous bone of implants in the iliac crest of sheep suffered peri-implant bone loss after being heated to 50 °C for 1 min.^23^ At temperatures around 60 °C lasting for 1 min, the peri-implant bone loss influenced implant osseointegration.^23^ Conversely, investigating heat treatment of the cortical sheep mandible at 50 °C for 1 min revealed no bone resorption and no threshold for heat-induced injury.^24^ The latter authors concluded that low bone density is more subject to heat-induced injury. Depending on gender, age, physique, time of edentulism, and pre-existing conditions, bone quality structures vary greatly in size.^25^ The mandible bone is also known to present a harder structure than the maxilla because of its thicker amount of cortical bone and because the greater part of the maxilla is composed of cancellous bone. However, in the present study, no significant correlation was evaluated between the bone quality of the upper or lower jaw and either osteocyte condition or matrix degeneration.

A critical reflection on the present study reveals that, in a cadaveric model, no blood flow was present. Therefore, defining reliable threshold temperature values requires in vivo investigations. However, the current study confirmed that treatments of 51 °C for 10 s and 5 °C for 30 s significantly led to matrix degeneration. According to Fajardo et al., in bony gaps filled with fluid (such as blood), the thermal conductivity of trabecular bone increases.^26^ Neither the maximum temperature nor the position of the isotherms was affected by the presence of fatty tissue or red marrow.^27^

By contrast, the EDX measurements confirmed that temperature substantially affected the Ca/C and Ca/P ratios. EDX is a sensitive, qualitative, and semiquantitative technique for assessing mineral content variations in microscopic regions of bone, and it has a spatial resolution of several cubic micrometers. It can detect calcification as an accumulation of calcium salts in a body tissue, which normally occurs during bone formation.^28^ The Ca/C ratio gives information concerning the degree of calcification in the bone matrix at respective measurement points,^29^ whereas the Ca/P ratio positively relates to induced bone loss^30^ as it indicates the presence of a basic calcium salt in freshly deposited bone salts. The Ca/P concentration ratio increases, while the C/Ca and C/P ratios decrease, in the healing bone matrix. In the present assessment, the measurements of the background indicated decreasing ratios for both Ca/P and Ca/C, while the debris areas increased. Therefore, temperature inputs, starting at 53 °C, may lead to decalcification.

Temperatures at 53 °C also induced mitochondrial swelling and a loss of the inner cristae structure. With increasing temperature, osteocytes showed typical signs of cellular stress. At 56 °C, the lumina of the endoplasmic reticulum were pronouncedly dilated, which is a typical endoplasmic reticulum reaction to stress.^31^

The purpose of this pre-clinical pilot study was to lay a foundation for future in vitro and in vivo studies focused on the potential use of thermally induced osteonecrosis for dental implant removal. Too many possible temperature/time intervals with different devices for warm and cold temperatures would lead to a huge sample size and increased recourse consumption. This pilot study was conducted to reduce these sample sizes in further, planned animal studies. Based on the temperature/time intervals of this pilot study, a subsequent thermo-explantation study in rats, with in vivo blood flow, will be carried out. This rat study will be feasible, as temperature/time levels of 51 °C for 10 s and 5 °C for 30 s presented significant matrix degeneration. Finally, successful thermo-explantation can only be validated on osseointegrated implants. Based on the rat study, another animal study in the pig with osseointegrated implants is planned.

## Conclusions

Though this is a preliminary study, the results identified temperatures and intervals, in the areas of both heat and cold, to lower the number of samples in further studies of temperature-induced bone necrosis. Temperature/time levels of 51 °C for 10 s and 5 °C for 30 s presented significant matrix degeneration. These levels may be used for future thermo-explantation. The matrix degeneration was evaluated as more severe at the same temperature and time intervals as the osteocyte condition. Furthermore, bone fraying did not appear to be caused by the temperature input. The subsequent planned in vivo study in rats, which will take into account the numeric parameters and blood flow, is feasible.

## References

[1] Derks J, Hakansson J, Wennstrom JL, Tomasi C, Larsson M, Berglundh T (2015). Effectiveness of implant therapy analyzed in a Swedish population: early and late implant loss. J Dent Res.

[2] Stajcic Z, Stojcev Stajcic LJ, Kalanovic M, Dinic A, Divekar N, Rodic M (2016). Removal of dental implants: review of five different techniques. Int J Oral Maxillofac Surg.

[3] Albrektsson T, Canullo L, Cochran D, De Bruyn H (2016). “Peri-implantitis”: a complication of a foreign body or a man-made “disease”. Facts and fiction. Clin Implant Dent Relat Res.

[4] Stajcic Z, Stojcev Stajcic LJ, Kalanovic M, Dinic A, Divekar N, Rodic M (2015) Removal of dental implants: review of five different techniques. Int J Oral Maxillofac Surg 10.1016/j.ijom.2015.11.003 26688293

[5] Anitua E, Murias-Freijo A, Alkhraisat MH (2016). Conservative implant removal for the analysis of the cause, removal torque, and surface treatment of failed nonmobile dental implants. The Journal of oral implantology.

[6] Bowkett A, Laverty D, Patel A, Addy L (2016). Removal techniques for failed implants. Br Dent J.

[7] Worni A, Marchand L, Sailer I, Cornish D, Hicklin SP (2018). Explantation of an osseointegrated titanium implant using laser-induced thermo-necrosis: a case report. Int J Oral Maxillofac Implants.

[8] Massei G, Szmukler-Moncler S. (2004) Thermo-explantation. a novel approach to remove osseointegrated implants. *European Cells and Materials Vol 7 Suppl 2, (page 48) ISSN 1473–2262*

[9] Cunliffe J, Barclay C (2011). Removal of a dental implant: an unusual case report. Journal of Dental Implants.

[10] Rouiller C, Majno G (1953). Morphological and chemical studies of bones after the application of heat. Beitrage zur pathologischen Anatomie und zur allgemeinen Pathologie.

[11] Lundskog J (1972). Heat and bone tissue. An experimental investigation of the thermal properties of bone and threshold levels for thermal injury. Scand J Plast Reconstr Surg.

[12] Eriksson AR, Albrektsson T (1983). Temperature threshold levels for heat-induced bone tissue injury: a vital-microscopic study in the rabbit. J Prosthet Dent.

[13] Goetz JE, Robinson DA, Pedersen DR, Conzemius MG, Brown TD (2011). Cryoinsult parameter effects on the histologically apparent volume of experimentally induced osteonecrotic lesions. J Orthop Res.

[14] Goetz JE, Pedersen DR, Robinson DA, Conzemius MG, Baer TE, Brown TD (2008). The apparent critical isotherm for cryoinsult-induced osteonecrotic lesions in emu femoral heads. J Biomech.

[15] Fondi C, Franchi A (2007). Definition of bone necrosis by the pathologist. Clin Cases Miner Bone Metab.

[16] Jansen P, Mumme T, Randau T, Gravius S, Hermanns-Sachweh B (2014). Endoglin (CD105) expression differentiates between aseptic loosening and periprosthetic joint infection after total joint arthroplasty. Springerplus..

[17] Faul F, Erdfelder E, Lang AG, Buchner A (2007). G*power 3: a flexible statistical power analysis program for the social, behavioral, and biomedical sciences. Behav Res Methods.

[18] Faul F, Erdfelder E, Buchner A, Lang AG (2009). Statistical power analyses using G*power 3.1: tests for correlation and regression analyses. Behav Res Methods.

[19] Prion S, Haerling KA (2014). Making sense of methods and measurement: spearman-rho ranked-order correlation coefficient. Clinical Simulation In Nursing.

[20] Wilcox CW, Wilwerding TM, Watson P, Morris JT (2001). Use of electrosurgery and lasers in the presence of dental implants. Int J Oral Maxillofac Implants.

[21] Eriksson A, Albrektsson T, Grane B, McQueen D (1982). Thermal injury to bone. A vital-microscopic description of heat effects. Int J Oral Surg.

[22] Mohlhenrich SC, Modabber A, Steiner T, Mitchell DA, Holzle F (2015). Heat generation and drill wear during dental implant site preparation: systematic review. Br J Oral Maxillofac Surg.

[23] Trisi P, Berardini M, Falco A, Vulpiani MP (2015). Effect of temperature on the dental implant osseointegration development in low-density bone: an in vivo histological evaluation. Implant Dent.

[24] Trisi P, Berardini M, Falco A, Vulpiani MP, Masciotra L (2014). Effect of 50 to 60 degrees C heating on osseointegration of dental implants in dense bone: an in vivo histological study. Implant Dent.

[25] Di Stefano DA, Arosio P, Pagnutti S, Vinci R, Gherlone EF (2019). Distribution of trabecular bone density in the maxilla and mandible. Implant Dent.

[26] Fajardo JE, Carlevaro CM, Vericat F, Berjano E, Irastorza RM (2018). Effect of the trabecular bone microstructure on measuring its thermal conductivity: a computer modeling-based study. J Therm Biol.

[27] Reznikov N, Shahar R, Weiner S (2014). Bone hierarchical structure in three dimensions. Acta Biomater.

[28] Murshed M (2018) Mechanism of Bone Mineralization. *Cold Spring Harbor perspectives in medicine.* 8(12) 10.1101/cshperspect.a031229 PMC6280711 29610149

[29] Okata H, Nakamura M, Henmi A, Yamaguchi S, Mikami Y, Shimauchi H, Sasano Y (2015). Calcification during bone healing in a standardised rat calvarial defect assessed by micro-CT and SEM-EDX. Oral Dis.

[30] Kourkoumelis N, Balatsoukas I, Tzaphlidou M (2012). Ca/P concentration ratio at different sites of normal and osteoporotic rabbit bones evaluated by auger and energy dispersive X-ray spectroscopy. J Biol Phys.

[31] Oslowski CM, Urano F. (2011) Chapter Four - Measuring ER stress and the unfolded protein response using mammalian tissue culture system. In: Conn PM, ed. *Methods in Enzymology.* Vol 490. Academic Press:71–92 10.1016/B978-0-12-385114-7.00004-0 PMC3701721 21266244

